# *Autographa californica* multiple nucleopolyhedrovirus *ac75* is required for egress of nucleocapsids from the nucleus and formation of *de novo* intranuclear membrane microvesicles

**DOI:** 10.1371/journal.pone.0185630

**Published:** 2017-10-02

**Authors:** Ya-Jun Guo, Shi-Hui Fu, Lu-Lin Li

**Affiliations:** Hubei Key Laboratory of Genetic Regulation and Integrative Biology, College of Life Sciences, Central China Normal University, Wuhan, China; Wuhan Bioengineering Institute, CHINA

## Abstract

In this study, Autographa californica multiple nucleopolyhedrovirus *ac75* was functionally characterized. *Ac75* has homologs in all sequenced genomes of alphabaculoviruses, betabaculoviruses, and gammabaculoviruses. It was determined to encode a protein that is associated with the nucleocapsid of budded virus and with both envelope and nucleocapsids of occlusion-derived virus. Sf9 cells transfected by an *ac75*-knockout bacmid resulted in the infection being restricted to single cells. No budded virus were detected although viral DNA replication and late gene expression were unaffected. Electron microscopy revealed that the virogenic stroma, nucleocapsids and occlusion bodies appeared normal in the cells transfected by an *ac75*-knockout bacmid. However, the nucleocapsids were unenveloped, the occlusion bodies did not contain any virions or nucleocapsids, and no nucleocapsids were found outside the nucleus or spanning the nuclear membrane. In addition, *de novo* intranuclear membrane microvesicles that are the precursor of occlusion-derived virus envelopes were absent in the nuclei of transfected cells. Confocal microscopy showed that AC75 protein appeared in the cytoplasm as early as 6 hours post infection. It localized to the ring zone at the periphery of the nucleus from 15 to 24 hours post infection and demonstrated light blocky cloud-like distribution in the center of the nucleus. AC75 was found to co-immunoprecipitate with BV and ODV associated envelope protein ODV-E25. The data from this study suggest that *ac75* is essential for induction of the intranuclear membrane microvesicles, it appears to be required for the intranuclear envelopment of nucleocapsids, and is also essential for egress of nucleocapsids from the nuclei, in infected cells.

## Introduction

*Autographa californica multiple nucleopolyhedrovirus* (AcMNPV) is the type species of the *Alphabaculoviruses*. It contains a genome of 134 kbp, with approximately 150 predicted protein-coding open reading frames (ORFs) [1, 2]. More than 110 of the AcMNPV ORFs have been characterized. Out of these characterized ORFs, forty-six encode proteins associated with virions or occlusion bodies; twenty-five are involved in viral DNA replication and/or gene expression; and fifteen encode proteins playing auxiliary roles during viral replication and infection, involving assembly, transport and release of progeny viruses, helping the virus traverse physical or physiological barriers, overcome host immune defenses, or affect the behavior of infected insects. Some genes encode proteins that are associated with virions and are also involved in DNA replication/gene expression or other auxiliary processes. Although the subject of intense research, the function of many AcMNPV genes are still unclear [2].

AcMNPV produces two types of structurally and functionally divergent virions, budded virus (BV) and occlusion-derived virus (ODV), during infection. BV and ODV are responsible for systemic infection within the body of infected host insects and to spread infection between host insects, respectively. The protein composition of AcMNPV BV and ODV has been determined by proteomics analysis and other studies [3, 4].

Although information is available on most of the AcMNPV orfs, the protein encoded by Ac75 has yet to be characterized. Homologs of *ac75* have been found in all sequenced genomes of alphabaculoviruses, beltabaculoviruses and gammabaculoviruses, but not deltabaculoviruses. A homolog of *ac75* in Bombyx mori NPV (BmNPV), *bm61*, was shown to be essential for virus replication, and the BM61 protein was shown to be associated with both BV and ODV[5, 6]. In Helicoverpa armigera nucleopolyhedrovirus (HearNPV), the homolog of AC75 (HA69) localized to the envelope of ODV and between the capsid and envelope of BV [7]. To investigate the role of *ac75* in the replication cycle of AcMNPV, mutants lacking *ac75* were constructed, transfected into Sf cells, and characterized to evaluate the effects of *ac75* deletion on BV production, viral DNA replication, late gene expression… The time course of AC75 expression, its subcellular location, and location in virions was also analyzed. It was found that *ac75* is required for induction of intranuclear membrane microvesicles and production of both BV and ODV.

## Materials and methods

### Virus, cell line and primers

The Sf9 cell line (Invitrogen Life Technologies) was cultured at 27°C in Grace's medium containing 10% fetal bovine serum, penicillin (100 μg/ml) and streptomycin (100 μg/ml). The AcMNPV bacmid bMON14272 derived from the AcMNPV strain E2 was maintained in DH10B cells as described previously [8].

The DNA primers used in the experiments are listed in Table 1

**Table 1:** Oligonucleotides used in this study.

Name	Sequence^a^	
ac75koFP	5’-CAAAAATACCAGCTCAATAGATTGATAAGTGTTTGTTAAACTTCGAATAAATACCTGTG-3’	
ac75koRP	5’-AACAATGCATGTATTAAAAAATCAACCTGTCGCCTACTGAAACCAGCAATAGACATAAG-3’	
ac75UP	ACGCGAGATTTATTCACGAAACCCG	
ac75DN	TTTTAAACCGTCCACCGTGTATTCG	
catPF	CGAATAAATACCTGTGACGGAAGAT	
catPR	GAAAACGGTGTAACAAGGGTGAAC	
ac75PF	GCGCTGCAGAATGTCCAATTTAATG	Pst I
ac75PR	GCGAAGCTTTTAATACGCTGGCAGTT	Hind III
P_ac75_PF1	5′-GCGGAATTCGTTTATTATCGTCAGC-3′	EcoR I
P_ac75_PF2	GGCTCTAGAGCTGTTGTACTATCGC(L)	Xba I
P_ac75_PR	ATCCGCTCGAGTTTTAACAAACACT	Xho I
ac75PR1	5′-AACCTCGAGTTAATACGCTGGCAGT-3′	Xho I
ac75PR2	5′-CATCTGCAGTTAATACGCTGGCAGT-3′	Pst I
ac75PR3	TATAGGATCCATACGCTGGCAGTTG(lack TAA)	BamH I
gfpPF	CGCGGATCCGTGAGCAAGGGCGAGGAGC	BamH I
gfpPR	GCGCTCGAGTTACTTGTACAGCTCGTC	Xho I
gfpPR1	CGGCCTGCAGTTACTTGTACAGCTCGTC	Pst I
ha-ac75PR	CTCGAGTTAGGCGTAATCTGGGACGTCGTATGGGTAATACGCTGGAGTTG	Xho I
M13F	GTTTTCCCAGTCACGAC	
M13R	CAGGAAACAGCTATGAC	
Q-65972F	CGTAGTGGTAGTAATCGCCGC	
Q-66072R	AGTCGAGTCGCGTC GCTTT	

^a^ The restriction site sequences are underlined.

### Antibodies and antibody preparation

The antisera against AC75 and GP41 were produced by the Core Facility Center, Wuhan Institute of Virology, Chinese Academy of Sciences. The anti-AC75 antiserum was prepared as below: The AcMNPV orf75 was amplified as a Pst I-Hind III fragment with primer pair ac75PF/ac75PR and inserted into the correspondent sites of pPROEx HTa (Invitrogen) to construct pPRO-ac75. *E*. *coli* BL21 (DE3) cells transformed with pPRO-ac75 was induced with IPTG. The His-tagged AC75 protein was separated by SDS-PAGE, and the band of the His-tagged AC75 protein was cut from the gel, homogenized and used to immunize a rabbit. Two weeks after the first inoculation, the rabbit was subjected to three boosts at 2-week intervals. 10 days after the final boost, it was bled and the serum was prepared. The anti-GP41 mouse antiserum was raised with GST-tagged GP41. The anti-E25, anti-VP39 and anti-E18 rabbit antisera were separately raised with corresponding His-tagged AcMNPV proteins previously [9, 10]. HA tag-specific monoclonal antibody was purchased from Proteintech^TM^.

### Western blot analysis

The protein samples were separated by 12% SDS-polyacrylamide gel electrophoresis (PAGE) and transferred to BioTace PVDF membrane (PALL Life Science). The blots were probed with individual specific antisera. IRDye-800CW conjugated goat anti-rabbit (or mouse) antibody (1:8,000) (LI-COR) was used as the secondary antibody. Fluorescence was detected by LI-COR Odyssey. SDS-PAGE and immunohybridization to western blots were performed in accordance with standard protocols and manufacturer’s instruction [11].

### Virion purification and fractionation

BV and ODV purification and subsequent fractionation into envelope and nucleocapsid was performed as previously described [9, 12]. Briefly, Sf9 cells were infected with AcMNPV at an MOI of 0.1, and the cells and medium were harvested separately at 5 days post infection. Eighty milliliters of the supernatant was centrifuged to remove cell debris. The supernatant was transferred to a fresh tube and centrifuged at 100, 000 g for 90 min to pellet BVs. The BV pellet was resuspended in 500 μl of 0.1×TE buffer and loaded onto a 20, 30, 40, 50 and 60 percent (w/v) discontinuous sucrose gradient, and centrifuged at 100,000 *g* (MLS50 rotor) for 90 min. The virus fraction was collected, diluted with four volumes of 0.1×TE, and centrifuged at 100,000 *g* for 90 min. The pellet was resuspended in 200 μl of 0.1× TE and stored at -20°C. For fractionation, 170 μl of the BV sample was mixed with 800 μl of 2.0% NP-40/10 mM Tris (pH8.5) and incubated at room temperature for 60 min with gentle agitation. The solution was layered onto a 4-ml 30% (v/v) glycerol/10 mM Tris (pH8.5) cushion and centrifuged at 150,000 g for 60 min (MLS 50 rotor). The nucleocapsid pellet was washed once with 0.1× TE. Following centrifugation at 150,000 g for 45 min, the pellet was resuspended in 70 μl of 0.1× TE. The envelope fraction on the top of the gradient was collected and mixed with 4 volumes of ice-cooled acetone. The mixture was centrifuged at 150,000 g for 45 min. The protein precipitates were washed once with ice-cooled acetone. The pellet was dissolved in 60 μl of 10 mM Tris (pH 7.4).

For ODV purification, the cell pellet from the infected cell cultures (200 ml) was resuspended in 15 ml of 0.2% Triton-X100 and subjected to sonication. The cell lysates were diluted with 0.2% Triton-X100 to 60 ml, then, layered onto a 30% sucrose/0.2% Triton-X100 cushion and centrifuged at 9,000 rpm (JS-24 rotor) for 20 min. The pellet was resuspended in 5 ml of 0.2% Triton-X100, loaded onto a 33.5 ml 35–60% sucrose gradient (w/v) in H_2_O and centrifuged at 100,000 *g* for 30 min. The OB fraction was collected and washed twice by suspension in H_2_O and centrifugation. The OB pellet was resuspended in 2 ml of H_2_O, mixed with 3 ml of 3 × OB lysis buffer [9] and incubated at 37°C for 40 min, then, centrifuged at 500 g for 5 min. The supernatant was layered onto a 20, 30, 40, 50 and 60% (w/v) discontinuous sucrose gradient and centrifuged at 50,000 *g* (Beckman JS-24 rotor) for 30 min. The virus fraction was collected, washed by dilution in 3 volumes of 0.1× TE, centrifuged at 50,000 *g* (Beckman JM-24 rotor) for 60 min. The ODV pellet was resuspended in 200 μl of 0.1× TE. Fractionation of ODV was done in the same way as fractionation of BV. All the centrifugation steps were done at 4°C.

Twenty microlilters of individual samples of BV, ODV, envelope and nucleocapsid fractions were loaded and run on 12% SDS-PAGE gel. Immunoblotting was performed as described above with polyclonal antibodies against AC75 (1:10,000), polyclonal antibodies against E25 (1:5000), or polyclonal antibodies against VP39 (1:10,000).

### Plasmid and bacmid construction

Construction of *ac75* knockout bacmids was done with the method described by Datsenko & Wanner [13]. Briefly, a DNA fragment containing the chloramphenicol acetyltransferase gene (*cat*) cassette flanked by two 40 bp homologous arms corresponding to the sequence (nt 63528–63567) immediately upstream of the start codon ATG and the 3’-end (nt 63259–63298) of AcMNPV *ac75* (nt 63126–63527) respectively was PCR-amplified with primers ac75koPF and ac75koPR. It was electro-transformed into arabinose-preinduced DH10B cells harboring bMON14272 and plasmid pKD46 encoding λ-Red recombinase. The white colonies growing on the plate with LB medium containing kanamycin, chloramphenicol and X-gal were selected. Four sets of primers, ac75UP/ac75DN, ac75UP/catPR, catPF/ ac75DN and catPF/catPR, were used in PCR to confirm the proper replacement of *ac75* with *cat* cassette in recombinants. The resultant bacmid was named vAc^ac75ko^. pFB-PH, a plasmid containing a copy of AcMNPV *polh* with native promoter and pFB-P_gp16_-egfp containing a copy of the enhanced green fluorescence protein gene (*egfp*) under control of AcMNPV *gp16* promoter [14] were transformed into *E*. *coli* DH10B containing vAc^ac75ko^ and pMON7124 to generate vAc^ac75ko-PH^ and vAc^ac75ko-gfp^, respectively. A fragment containing *ac75* with its native promoter (nt 63126–63711) was PCR-amplified using primers P_ac75_PF1 and ac75PR1 and inserted between the Bst1107 I and Xho I sites of pFD-*polh*, a plasmid constructed by inserting a copy of AcMNPV *polh* with native promoter between the Bst1107 I and Pst I, to obtain pFB-ac75-polh. Another PCR-amplified (with primers P_ac75_PF1 and ac75PR2) fragment containing *ac75* with its native promoter was inserted between the EcoR I and Pst I sites of pFD-P_gp16_-gfp [14] to make pFD-P_gp16_-gfp-ac75. pFB-ac75-polh and pFD-P_gp16_-gfp-ac75 were separately transformed into *E*. *coli* DH10B containing vAc^ac75ko^ and pMON7124 to generate ac75-repaired bacmid vAc^ac75ko-rep-PH^ and vAc^ac75ko-rep-gfp^, respectively.

Plasmids pP_acvp39_-luc, pP_acgp41_-luc, pP_acpk1_-luc, pP_acan_-luc, pP_acp6.9_-luc, pP_ace18_-luc and pP_acpolh_-luc, which separately contain the reporter gene (*luc*) linking with individual promoters of AcMNPV *vp39*, *gp41*, *p6*.*9*, *pk1*, *an*, *e18*, and *polh* [15], were transformed into *E*. *coli* DH10B containing vAc^ac75ko^ to generate reporter bacmids vAc^ac75ko-Pvp39-luc^, vAc^ac75ko-Pgp41-luc^, vAc^ac75ko-Pp6.9-luc^, vAc^ac75ko-Ppk1-luc^, vAc^ac75ko-Pan-luc^, vAc^ac75ko-Pe18-luc^, and vAc^ac75ko-Ppolh-luc^, and transformed into *E*. *coli* DH10B containing vAc^gp64ko^ to generate bacmids vAc^gp64ko-Pvp39-luc^, vAc^gp64ko-Pgp41-luc^, vAc^gp64ko-Pp6.9-luc^, vAc^gp64ko-Ppk1-luc^, vAc^gp64ko-Pan-luc^, vAc^gp64ko-Pe18-luc^,and vAc^gp64ko-Ppolh-luc^, respectively.

A fragment containing *egfp* ORF was PCR-amplified with primers gfpPF and gfpPR and inserted between the BamH I and Xho I sites of pFastBac-Dual (Invitrogen). The resultant plasmid was cut with BamH I and Xba I, and ligated with a Xba I-BamH I fragment containing AcMNPV *ac75* with native promoter and without stop codon (nt 63123–63849), which was amplified with primers P_ac75_PF2 and ac75PR3, to make pFD-ac75-gfp. The *ac75-egfp* fusion chimera was isolated from pFD-ac75-gfp by digestion with Xho I and Xba I and ligated with pFB-PH linearized with the same enzymes to generate pFB- ac75-gfp-polh. A PCR fragment comprising *ac75* promoter (nt63528-63849) was PCR-amplified with P_ac75_PF2 and P_ac75_PR, and inserted between the Xho I and Xba I sites and upstream of the *egfp* of an intermediate plasmid, which was made by inserting an *egfp* into pFastBac-dual, resulted in pFD-P_ac75_-gfp. pFB-ac75-gfp-polh and pFB-P_ac75_-gfp was transformed into DH10B cells containing vAc^ac75ko^ and DH10B cells containing bMON14272, respectively, to produce reporter bacmids vAc^ac75-gfp-PH^ and vAc^Pac75-gfp^.

A PCR fragment containing *ac75* fused with a HA-tag coding sequence at its 3’-end was synthesized with primers P_ac75_PF2 and ha-ac75PR and inserted between the Xba I and XhoI sites of pFastBac-dual to generate pFD-ac75-HA. pFD-ac75-HA was transformed into DH10B cells containing vAc^ac75ko^ to produce vAc^ac75HA^.

All the transfer vectors above were sequenced to confirm the construction. All of the bacmid constructs made by transposition were confirmed by PCR with primer set M13F/R (Table 1).

A DNA fragment containing *ac75* fused with *egfp* was amplified from pFD-ac75-gfp with primers ac75PF3 and gfpPR1, digested with Pst I, then ligated to a linearized intermediate plasmid pP_ie1_-pxie1 that contains an AcMNPV *ie1* promoter and a *p10* transcription termination signal sequence, replacing the pxie1 and generate reporter plasmid pP_ie1_-ac75-gfp.

### Titration of BV

For titering of infectious BV, Sf9 cells in 35-mm plates were inoculated with the supernatant from transfection with appropriate bacmid, at an MOI of 5. The supernatants were collected at various time points post infection and used to inoculate fresh cell cultures in a 96-well plate, and the BV titers were determined using a TCID_50_ end-point dilution assay [16]. Virus infection was determined by viewing green fluorescence from EGFP expressed by the viruses.

Tittering of total BVs released from transfected cells was performed as previously described [9]. Sf9 cells seeded in 35-mm plates (1×10^6^ cells/plate) were transfected with vAc^PH^ or vAc^ac75ko-PH^. At designated time points, the supernatants were collected and centrifuged at 3,000 rpm for 7 min to remove cells, then, transferred to fresh tubes and centrifuged at 7,000 rpm for 7 min. Individually, 500 μl of the supernatant was mixed with equal volume of the lysis buffer [9] and incubated at 50°C for 12 h. DNA was extracted with phenol-chloroform-isoamyl alcohol (25:24:1), followed by precipitation with isopropanol and centrifugation. The DNA pellet was dissolved in 10 μl of ddH_2_O. One microliter of each purified DNA sample was used as template for each qPCR reaction.

### Quantitative real-time PCR

Real-time PCR was done as previously described[17]. For analysis of viral DNA replicated in transfected cells, Sf9 cells seeded in 35-mm plates were transfected with vAc^ac75ko-PH^, vAc^ac75ko-rep-PH^, or vAc^PH^ were harvested at designated time points. The DNA was extracted by phenol-chloroform-isoamyl alcohol and precipitated with isopropanol. Each DNA pellet was dissolved in 80 μl of ddH_2_O. Seven point five microliters of DNA from each sample was treated with 4 units of Dpn I in a 10 μl of total reaction volume. One microliter of each Dpn I-digested DNA sample was used for each qPCR reaction.

The DNA purified from cells or supernatants from transfection was mixed with the SYBR-Green I Real-Time PCR Master Mix Kit (Toyobo) and the qPCR primers Q-65972F and Q-66072R [17] in a 20-μl reaction. The samples were analyzed in a Bio-Rad CFX96 qPCR cycler. The results were analyzed using CFX Manager 2.1 (Bio-Rad) software. The qPCR product corresponded to a 100-bp region of the AcMNPV *gp41* gene. The number of viral DNA genome copies within each sample was calculated by using a standard curve generated from a dilution series of a plasmid containing AcMNPV *gp41*.

### Luciferase assay

Sf9 cells seeded in wells of a 96-well tissue culture plate were transfected with individual reporter bacmids. At designated time points post-transfection, the medium was removed, the cell layers were rinsed three washes with phosphate-buffered saline, and the cells were lysed by the addition of lysis buffer (Promega, Madison, WI, USA). Luciferase activity was determined using Luciferase Activity Reagent (Promega), and the chemiluminescent reactions were measured using a CentroXS^3^ LB960 (Berthold Technologies, Zug, Switzerland).

### Electron microscopy

Sf9 cells seed in 35-mm plate (1×10^6^ cells/plate) were transfected with 2.0 μg of vAc^ac75ko-rep-PH^ or vAc^ac75ko-PH^. At 48 and 96 hpt, the cells were fixed with 2.5% glutaradehyde, then dislodged and collected by centrifugation at 2,400 rpm for 5 min. The cell pellet was dehydrated, embedded, sectioned, and stained as described previously [9]. Samples were examined with a FEI Tecnai G^2^20 TWIN transmission electron microscope at an accelerating voltage of 200 kV.

### Confocal microscopy

Sf9 cells seeded in 35-mm plates were infected with vAc^ac75-gfp-PH^ or vAc^Pac75-gfp^ (at an MOI of 10), or transfected with pP_ie1_-ac75-gfp (2 μg/plate). At designated time points, the cells were visualized with a ZEISS, LSM710 NLO confocal laser scanning microscope for fluorescence using a wavelength of 488 nm laser line for EGFP. All images were digitally recorded and merged by the use of ZEISS software.

### Immunoprecipitation

Immunoprecipitation assays were performed as previous descrbed [9]. Briefly, Sf9 cells infected with vAc^HA-ac75^ were harvested at 18, 24, 36 and 48 hours post infection (hpi) respectively. The cell pellets were rinsed with PBS, then, mixed and resuspended in 600 μl of lysis buffer, incubated on ice with shaking for 1 h. The lysates were clarified by centrifugation at 11, 000 rpm for 25 min. The supernatant (protein concentration was adjusted to 2 ng/ul) was mixed with the HA-specific monoclonal antibody (4 μg), rocked at 4°C for 1 h, then mixed with 40 μl of Protein A/G Plus beads (Santa Cruz Biotechnology) and rocked at 4°C for 6 h. The beads were precipitated and washed three times with cold lysis buffer, then mixed with 40 μl 2 x Laemmili buffer, denatured at 100°C, and precipitated. The supernatant was subjected to 12% SDS-PAGE and western-blot. The blots were probed with the individual antisera to E25 (1:10,000), E18 (1:10,000), GP41 (1:4000) or monoclonal antibody to HA-tag (1:3,000).

## Results

### AC75 is associated with both BV and ODV nucleocapsids and the ODV envelope

**Fig 1:**
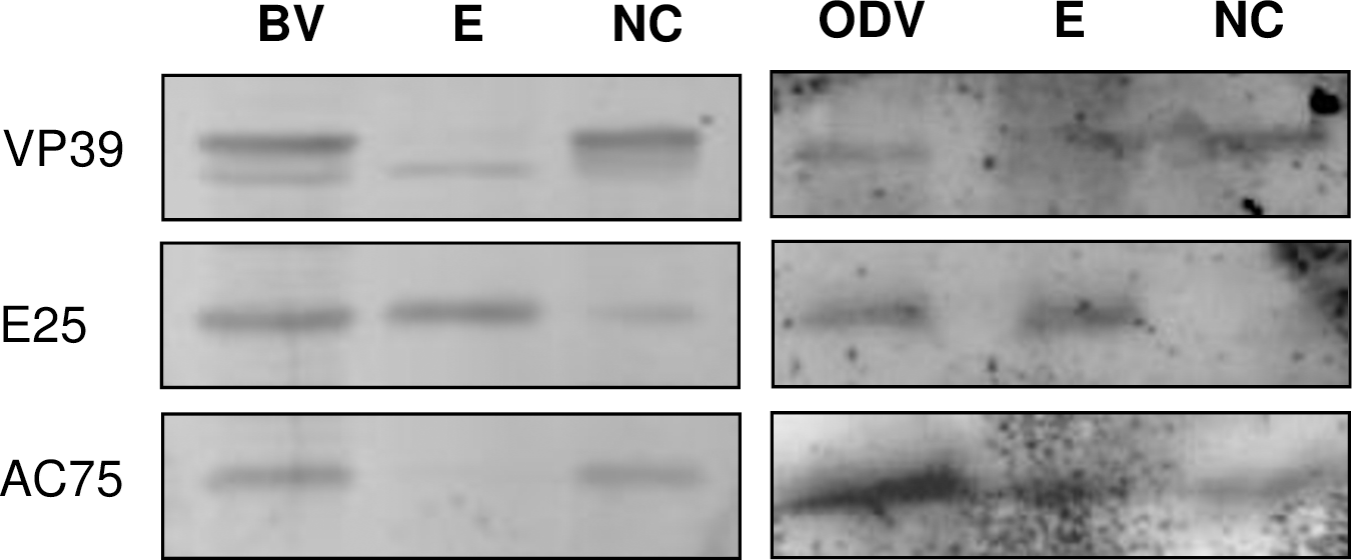
Analysis of AC75 in purified and fractionated BV and ODV. BV and ODV were purified by centrifugation using sucrose gradients and fractionated into envelope and nucleocapsid fractions, and then analyzed by 12% SDS-PAGE and western blotting. AC75 was probed with AC75-specific polyclonal antibodies. The nucleocapsid protein VP39 and envelope protein E25 were detected with corresponding specific antibodies. NC, nucleocapsid fraction; E, envelope fraction.

The *ac75* comprises 402 nt (133 aa) and encodes a predicted polypeptide of 15.5 kDa. Bioinformatics analysis revealed that it is related to a DUF1160 super family of proteins of unknown function. To determine whether it was a structural protein, BV and ODV were separately purified, fractionated into envelope and nucleocapsid fractions, and immunoblotted using AC75-specific antibodies. The nucleocapsid protein VP39 and envelope protein E25 were probed in identical samples, as controls. A polypeptide of the predicted size of AC75 was observed in the nucleocapsid fraction of BV and in both the nucleocapsid and envelope fractions of ODV. VP39 and E25, as expected, were detected in the nucleocapsid of BV and ODV and in the envelope fraction of ODV (Fig 1). Therefore Ac75 appears to be associated with both the BV and ODV nucleocapsids and the envelope of ODV.

### The *Ac75* knockout eliminated production of infectious virus

**Fig 2:**
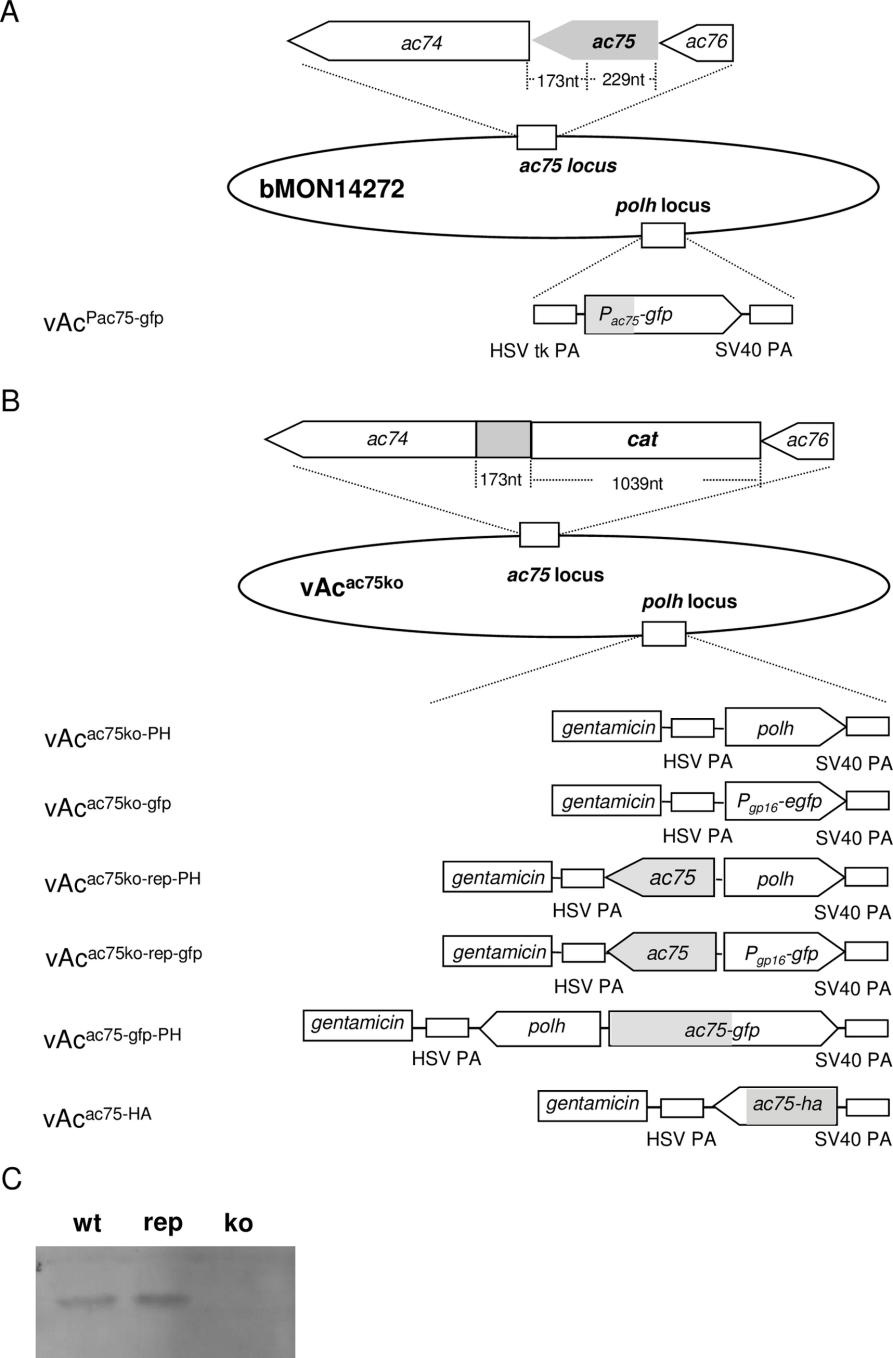
Construction of *ac75* knockout, repair and wt AcMNPV bacmids. (A) Maps showing the structure of the *ac75* locus in bMON14272, and the modification of the *polh* locus in a wt AcMNPV bacmids. A copy of *egfp* under control of the *ac75* promoter was inserted at the *polh* locus of bMON14272 to construct vAc^Pac75-gfp^. (B) Maps showing the modification of the *ac75* and *polh* loci in *ac75* knockout and repair AcMNPV bacmids. At the *ac75* locus, a 229 nt sequence of the *ac75* ORF was replaced with the *cat* gene (vAc^ac75ko^). At the *polh* locus the following constructs were produced: a copy of *egfp* under control of the *gp16* promoter (Pgp16-gfp) (vAc^ac75ko-gfp^), *polh* with its native promoter (vAc^ac75ko-PH^), or a copy of *ac75* with its native promoter and Pgp16-gfp (vAc^ac75ko-rep-gfp^), *ac75* with its native promoter and the *polh* gene with its native promoter (vAc^ac75ko-rep-PH^), *egfp* fused with *ac75* with its native promoter and a *polh* gene with its native promoter (vAc^ac75-gfp-PH^), or a copy of *ac75* with its native promoter fused with an HA-tag at its 3’-end (vAc^ac75-HA^). (C) Western blot analysis for the presence or absence of AC75 in extracts of the cells transfected by vAc^ac75ko-gfp^ (ko) or wild type AcMNPV (wt) or vAc^ac75ko-rep-gfp^ (rep).

In order to determine if *ac75* is essential for replication of AcMNPV, several mutants with the *ac75* knocked out, and *ac75*-knockout repaired were constructed. In the *ac75* knockout mutants, the first 229 nt at the 5’-end of *ac75* was replaced with the *cat* gene (Fig 2A and 2B), and a copy of the *polh* gene with its native promoter (vAc^ac75ko-PH^) or the *egfp* linked with a copy of AcMNPV *gp16* promoter (vAc^ac75ko-gfp^), a late promoter, was inserted at the *polh* locus to facilitate detection of viruses in cells (Fig 2B). The *ac75*-knockout repair mutants were constructed by inserting a copy of *ac75* together with a copy of *polh* (vAc^ac75ko-rep-PH^) or *egfp* (vAc^ac75ko-rep-gfp^) at the *polh* locus of the *ac75* knockout mutant. Deletion of *ac75* in the *ac75*-knockout mutants was confirmed by western-blot, using the AC75-specific antiserum. A peptide of about 15.5 kDa was detected in extracts of the cells transfected by vAc^ac75ko-rep-gfp^ or vAc^gfp^, but was not present in extracts of the cells transfected by vAc^ac75ko-gfp^ (Fig 2C).

**Fig 3:**
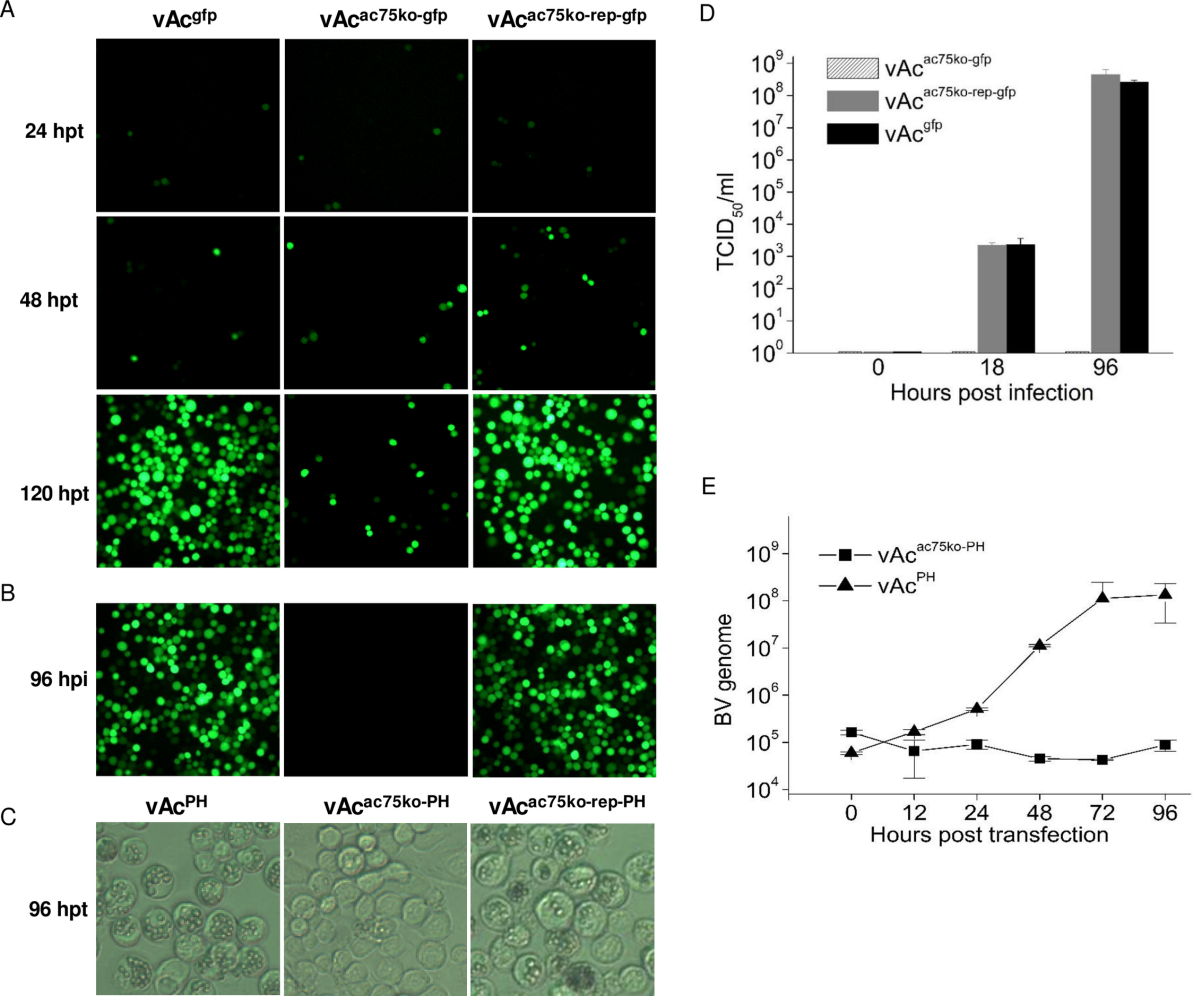
Analysis of BV production of the *ac75* knockout mutant in transfected cells. (A) Fluorescence microscopy of Sf9 cells transfected with vAc^gfp^, vAc^ac75ko-gfp^ and vAc^ac75ko-rep-gfp^ at designated time points post transfection. (B) Fluorescence microscopy of Sf9 cells inoculated with supernatants from transfections with vAc^gfp^ or vAc^ac75ko-gfp^ or vAc^ac75ko-rep-gfp^, at 96 hpi. (C) Light microscopy of Sf9 cells transfected with vAc^PH^ or vAc^ac75ko-PH^ or vAc^ac75ko-rep-PH^, at 96 hpt. (D) Titration of *ac75* knockout, repair, and wt viruses in Sf9 cells. Sf9 cells were infected with each virus at an MOI of 5, or inoculated with 1 ml of supernatant from the transfection with vAc^ac75ko-gfp^. The supernatants were harvested at 0, 18 and 96 hpi and the virus titers were determined by TCID_50_ end-point dilution assays. Each data point represents the average titer of three independent infections. Error bars indicate standard deviations. (E) BV growth curves of *ac75* knockout and wt bacmid-transfected Sf9 cells independent of viral infectivity. DNA was purified from the supernatants collected at the designated time points from the transfections and the titers were determined by qPCR. Error bars represent standard deviation from triplicate samples.

The effect of the deletion of the *ac75* on replication of the virus was first examined by fluorescence microscopy of the Sf9 cells transfected with either vAc^ac75ko-gfp^, vAc^ac75ko-rep-gfp^, or vAc^gfp^, a bacmid constructed by inserting an *egfp* under control of gp16 promoter [14]. The cells from each culture were viewed at various time points post transfection. As shown in Fig 3A, weak fluorescence was first observed in cells at 18 hours post transfection (hpt). At 24 hpt, more cells with bright fluorescence were present in all transfected cell cultures. By 120 hpt, the majority of the cells in the cultures with vAc^gfp^ or vAc^ac75ko-rep-gfp^ were fluorescent, while there was only a slight increase in the number of fluorescent cells in the culture transfected with vAc^ac75ko-gfp^ indicating that this construct was unable to infect adjacent cells. To test if infectious BVs were produced in the transfections, the supernatants collected from each transfection, at 120 hpt, were individually inoculated into fresh cultures of Sf9 cells. By 96 hpi, almost all cells in the plates inoculated with supernatant of vAc^gfp^ or vAc^ac75ko-rep-gfp^ were filled with fluorescence. In contrast, no fluorescent cells were observed in the culture inoculated with the supernatant from the transfection with vAc^ac75ko-gfp^ (Fig 3B). In cells transfected with *polh*-containing mutants, OBs were observed in most of the cells transfected with vAc^PH^ or vAc^ac75ko-rep-PH^, by 96 hpt, but they were found only in a few cells in the culture transfected with vAc^ac75ko-PH^, in which the OB-containing cells were isolated, and the numbers of OB contained in the cells was obviously less than those in the cells with vAc^PH^ or vAc^ac75ko-rep-PH^ (Fig 3C). These observations demonstrated that the wt and the *ac75* knockout repair bacmid replicated in the transfected cells and produced infectious viruses, whereas no infectious BV was released from the cells transfected with the *ac75* knockout bacmid. However, the *ac75* knockout did not block formation of OBs.

The titers of BV released from the cells inoculated with 1 ml of supernatant from transfection with vAc^ac75ko-gfp^, or infected with vAc^ac75ko-rep-gfp^ or vAc^gfp^, at an MOI of 5, were measured at 0, 18 and 96 hpi, respectively by TCID_50_ end-point dilution assays. As shown in Fig 3D, whereas the titers of infectious BV sharply increased for both vAc^ac75ko-rep-gfp^ and vAc^gfp^, by 96 hpi, without significant difference between these two viral types, the virus titer from the cell culture inoculated with the supernatant from the transfection with vAc^ac75ko-gfp^ was undetectable. The results showed that the effect of knocking out *ac75* on production of infectious BV could be completely recovered by reinserting a copy of *ac75* at the *polh* locus.

To determine whether the *ac75* knockout affected production or infectivity of BV, titers of both infectious and noninfectious BVs released from transfected cells at selected time points post transfection were measured by qPCR. As shown in Fig 3E, there were detectable background levels of viral genomes present at all time-points analyzed due to genomic DNA present from the bacmid transfection. As expected, a steady increase in viral genomic DNA was detected from 24 to 96 hpt for vAc^PH^, resulting from the continuous release of BV from transfected or infected cells. For vAc^ac75ko-PH^, the quantity of viral genomic DNA detected was lower than the background level at all time points post transfection. This result demonstrated that there did not appear to be any BV released from the cells transfected with vAc^ac75ko-PH^.

### *ac75* knockout had no effect on virus DNA replication and late gene expression

**Fig 4:**
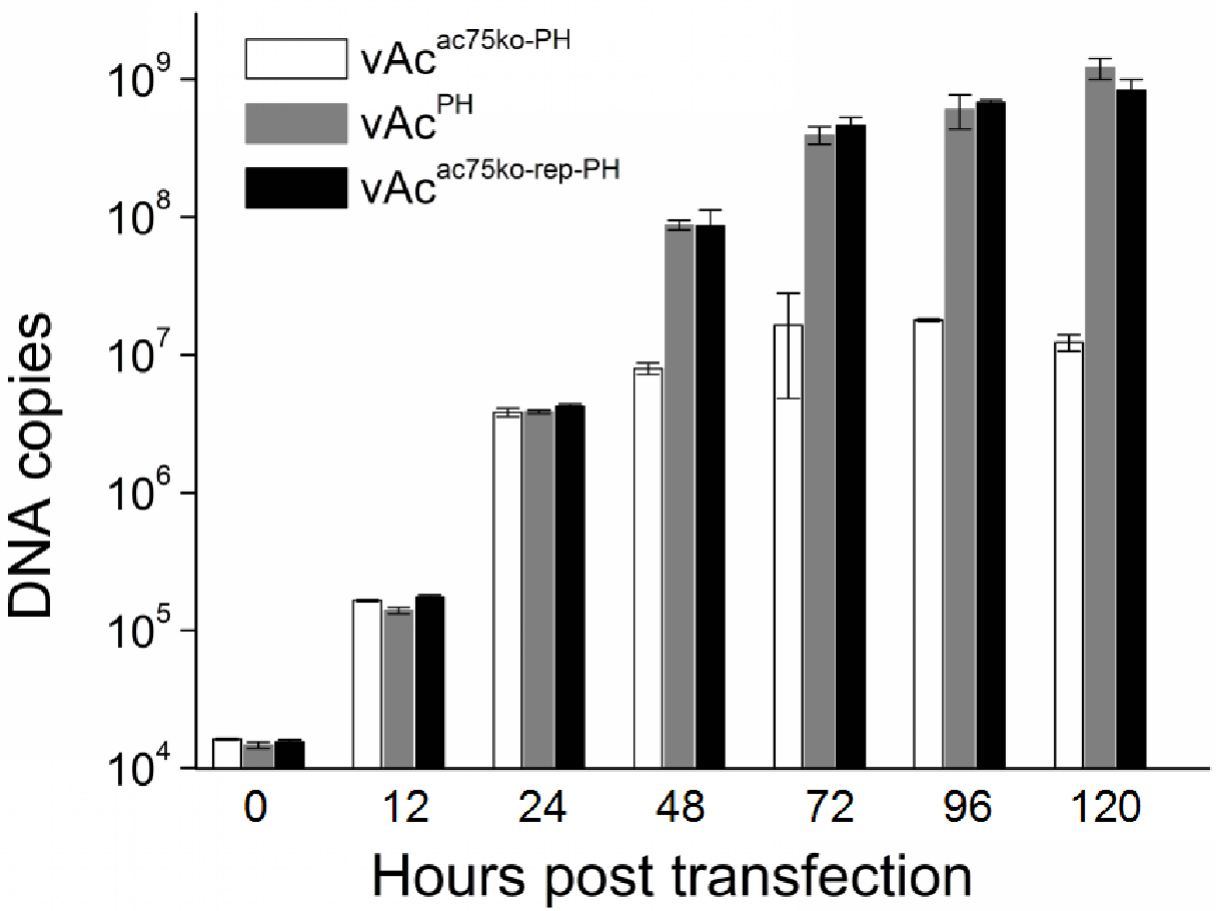
Quantitative-PCR analysis of viral DNA replication of the *ac75* knockout mutant in transfected cells. Total DNA was purified from the cells transfected with vAc^ac75ko-PH^, vAc^ac75ko-rep-PH^, or vAc^PH^ at 0, 6, 12, 24, 48, 72, 96 and 120 hpt, digested with Dpn I to eliminate input bacmid DNA, and the *gp41* sequence was analyzed by real-time PCR. The values displayed represent the averages from infections performed in triplicate with error bars indicating standard deviations.

To examine the effect that *ac75* knockout may have on viral DNA replication, Sf9 cells were transfected with vAc^ac75ko-PH^, vAc^ac75ko-rep-PH^, and vAc^PH^, respectively, collected at designated time points, and the total DNA was extracted and analyzed by qPCR. The results are shown in Fig 4. For all the three bacmids, DNA synthesis began increasing at 12 hpt, the levels of DNA detected for all the three bacmids were similar by 24 hpt. After that, the DNA levels measured for three bacmids rose continuously, but the levels of vAc^ac75ko-rep-PH^ and vAc^PH^ were much higher than that of vAc^ac75ko-PH^. The differences likely resulted from secondary infection of adjacent cells by the BVs released from the cells transfected by vAc^ac75ko-rep-PH^ or vAc^PH^ since vAc^ac75ko-PH^ could not produce infectious BV to infect adjacent cells. These results suggested that the deletion of *ac75* did not have any effect on viral DNA replication.

**Fig 5:**
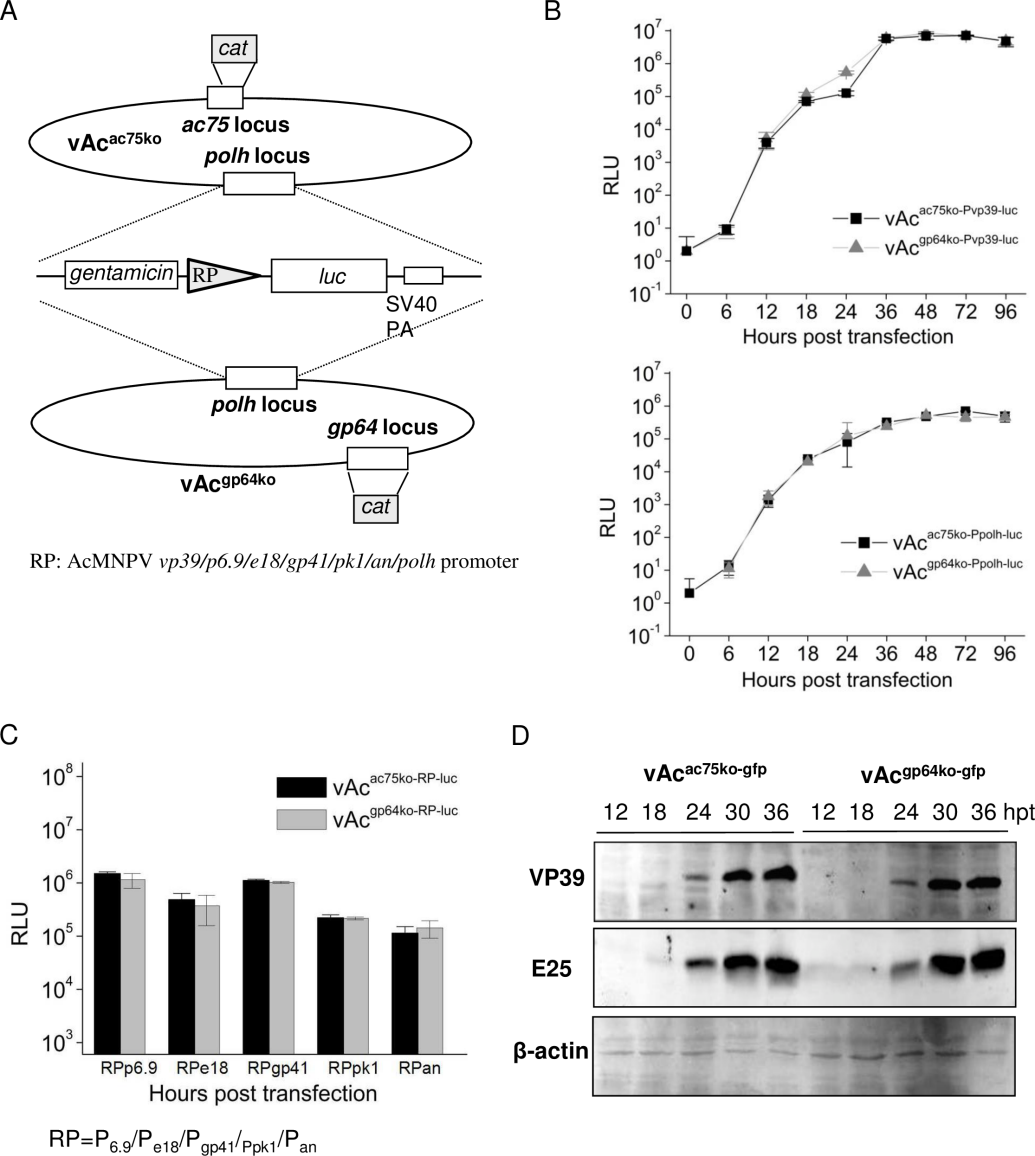
Analysis of late gene expression of the *ac75* knockout mutant in transfected cells. (A) Schematic maps of the structures of the reporter bacmids for late gene expression. A copy of *luc* under control of individual promoters of *vp39*, *p6*.*9*, *e18*, *gp41*, *pk1*, *an*, and *polh* was inserted into the *polh* locus of vAc^ac75ko-gfp^ or vAc^gp64ko-gfp^. (B) & (C) LUC assays of the extracts of the Sf9 cells transfected with the designated reporter bacmids for *vp39*, *p6*.*9*, *e18*, *gp41*, *pk1*, *an*, and *polh* in *ac75*-knockout or *gp64*-knockout mutants. LUC activities measured at 0, 6, 12, 18, 24, 36, 48, 72 and 96 hpt for promoter of *vp39* and *polh* (B) and the ones measured at 36 hpt for *p6*.*9*, *e18*, *gp41*, *pk1*and *an* (C) are shown. Error bars represent the standard error from three independent experiments. (D) Western blot analysis of VP39 and E25 synthesized in Sf9 cells transfected with vAc^ac75ko-gfp^ or vAc^gp64ko-gfp^. Transfected Sf9 cells were harvested at the designated times post infection, and cell lysates were prepared for western blot analysis. Each protein was analyzed with polyclonal antibodies against the specific protein. An anti-actin antibody was used as a loading control.

Effects of *ac75* knockout on virus late gene expression were evaluated by assays of reporter gene expression from the promoters of *vp39*, *p6*.*9*, *e18*, *gp41*, *pk1*, *an*, and *polh*, which are known late genes, and by western-blotting of the major capsid protein VP39 and envelope protein E25. For reporter gene expression assays, reporter bacmids vAc^ac75ko-Pvp39-luc^, vAc^ac75ko-Pe18-luc^, vAc^ac75ko-Pp6.9-luc^, vAc^ac75ko-Pgp41-luc^, vAc^ac75ko-Ppk1-luc^, vAc^ac75ko-Pan-luc^, and vAc^ac75ko-Ppolh-luc^, which contain a *luc* gene under control of the individual promoters as designation, were constructed (Fig 5A). All of the reporter bacmids have *ac75* deleted. Sf9 cells transfected with individual reporter bacmids were collected at designated time points and used for LUC assays. Cells transfected by reporter bacmids vAc^gp64ko-Pvp39-luc^, vAc^gp64ko-Pp6.9-luc^, vAc^gp64ko-Pe18-luc^, vAc^gp64ko-Pgp41-luc^, vAc^gp64ko-Ppk1-luc^, vAc^gp64ko-Pan-luc^, and vAc^gp64ko-Ppolh-luc^. The latter construct lacks the membrane fusion protein gene *gp64* and was used as a control. As shown in Fig 5B, the levels of LUC activity detected in extracts of cells transfected with vAc^ac75ko-Pvp39-luc^ or vAc^ac75ko-Ppolh-luc^ were similar to those transfected with vAc^gp64ko-Pvp39-luc^ or vAc^gp64ko-Ppolh-luc^ at all time points. Similar results were found in the assays with the other reporter bacmids. Fig 5C shows the LUC levels detected in the cells transfected with the individual reporter bacmids for *p6*.*9*, *e18*, *gp41*, *pk1* and *an*, at 36 hpt, and demonstrates no significant difference between the reporter bacmids with *ac75* knocked out and the reporter bacmids lacking *gp64*

In the western-blot assays, Sf9 cells transfected by vAc^ac75ko-gfp^ or vAc^gp64ko-gfp^ were harvested at 12, 18, 24, 30 and 36 hpt, and the cell lysates were analyzed. As shown in Fig 5D, the levels of VP39 and E25 detected in the cells transfected with vAc^ac75ko-gfp^ were similar to the ones in the cells with vAc^gp64ko-gfp^ at all time points. The results from both LUC assays and western-blot assays indicated that the *ac75* deletion did not have any detectable effect on viral late gene expression.

### Effects of *ac75* knockout on morphogenesis of the virus

**Fig 6:**
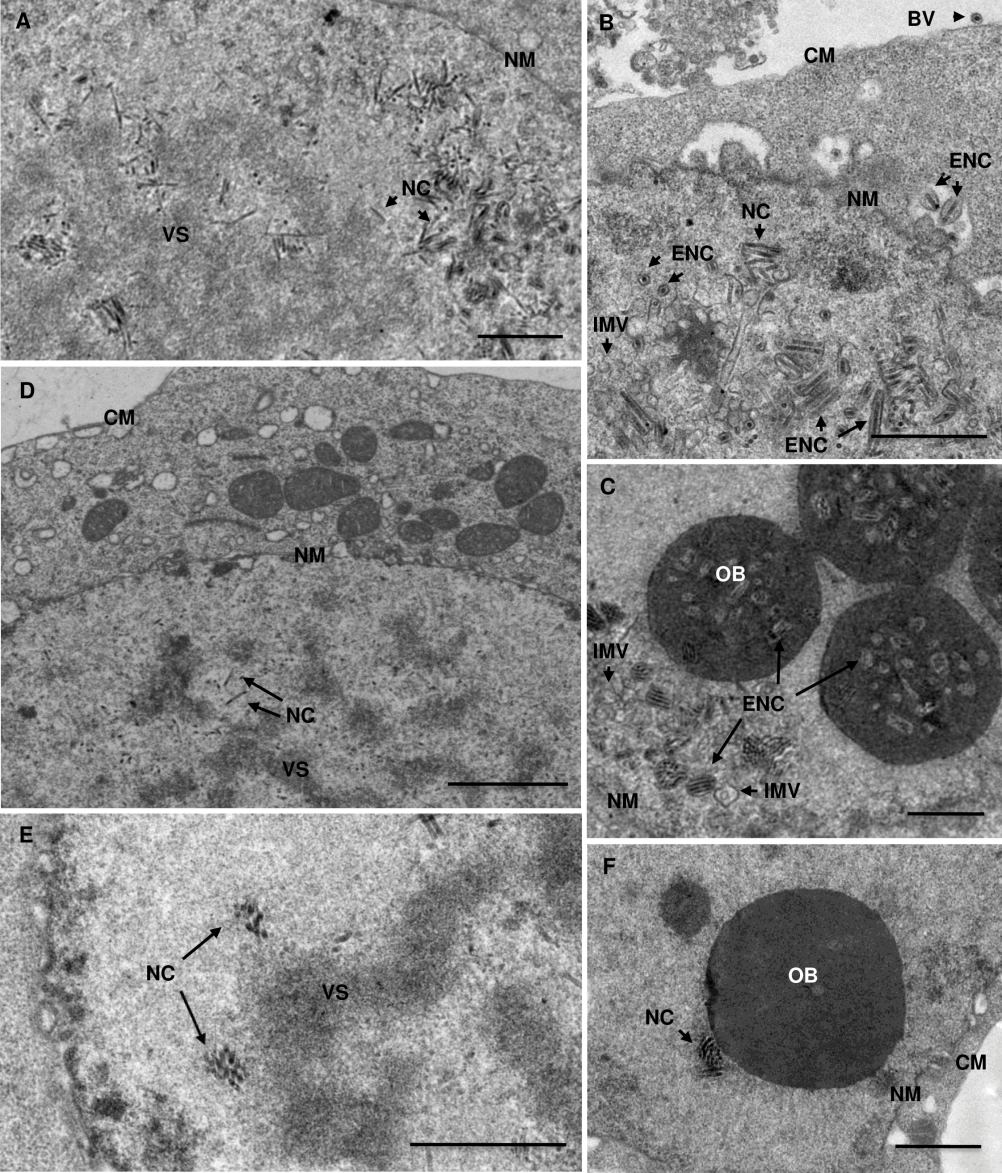
**Transmission electron microscopy of Sf9 cells transfected with vAc^ac75ko-rep-PH^ (A, B, C) or vAc^ac75ko-PH^ at 48 hpt (D) and 96 hpt (E, F)**. CM, cytoplasmic membrane; NM, nuclear membrane; NC, nucleocapsid; ENC, enveloped nucleocapsid; VS, virogenic stroma; IMV, intranuclear membrane microvesicles. Scale bar = 1μm.

Effects of *ac75* knockout on virus morphogenesis were examined by transmission electron microscopy of thin sections of the cells transfected with vAc^ac75ko-PH^ and the cells with vAc^ac75ko-rep-PH^. As shown in Fig 6A–6C, typical characteristics of baculovirus infection were viewed in the cells with vAc^ac75ko-rep-PH^. Virogenic stroma located in the center of nuclei had many nucleocapsids present (Fig 6A). In the peripheral regions of the nuclei, nucleocapsids accumulated and aligned with the nuclear envelope, and abundant intranuclear microvesicles were present (Fig 6B). Enveloped nucleocapsids released from the nucleus, and BVs released from the cells were observed (Fig 6B). OBs formed in the nuclei and contained multiple enveloped nucleocapsids (Fig 6C). In the cells transfected by vAc^ac75ko-PH^, virogenic stroma, nucleocapsids and OBs were seen in the nuclei but none of the nucleocapsids were enveloped and no enveloped virions or nucleocapsids were seen in OBs, and intranuclear microvesicles were not observed (Fig 6D, 6E & 6F). These phenotypes indicate that *ac75* knockout prevents the production of intranuclear microvesicles, which are required for envelopment of nucleocapsids.

### Expression time course and subcellular localization of AC75 in AcMNPV- infected Sf9 cells

**Fig 7:**
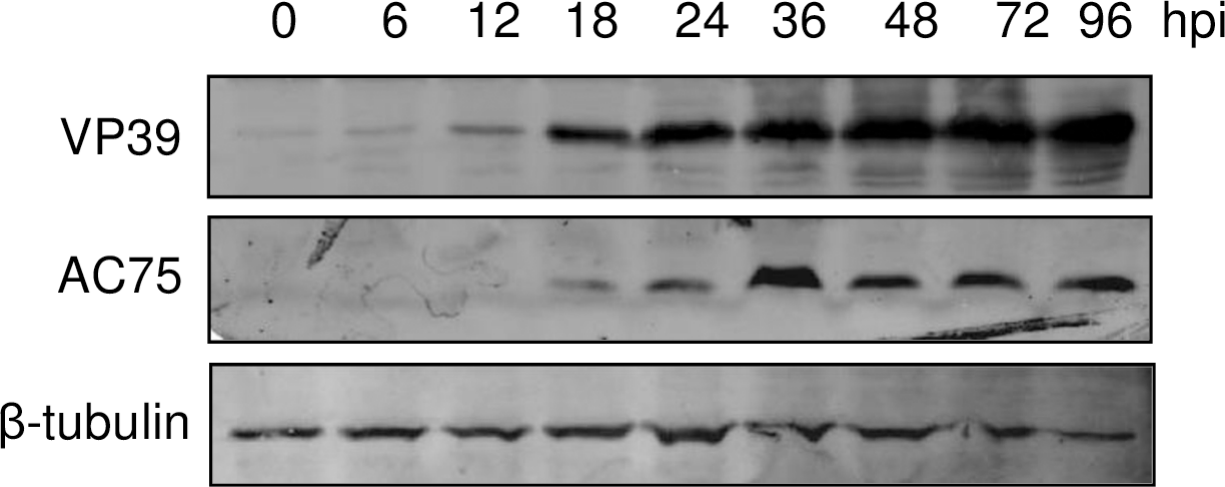
Time course analysis of AcMNPV AC75 expressed in infected Sf9 cells. The cells inoculated with infectious cell culture supernatant containing AcMNPV were harvested at designated time points post infection, and the cell extracts subjected to SDS-PAGE, and immunoblot analysis with AC75-specific and VP39-specific antisera.

To investigate the temporal expression of AC75, the time course of accumulation of the protein in AcMNPV-infected cells were examined by using the AC75-specific antiserum (Fig 7). As controls, β-tubulin of the cells and the viral VP39 in the same samples were also examined. AC75 was first detected at 18 hpi and the peak level of accumulation occurred at 36 hpi, consistent with late gene expression.

**Fig 8:**
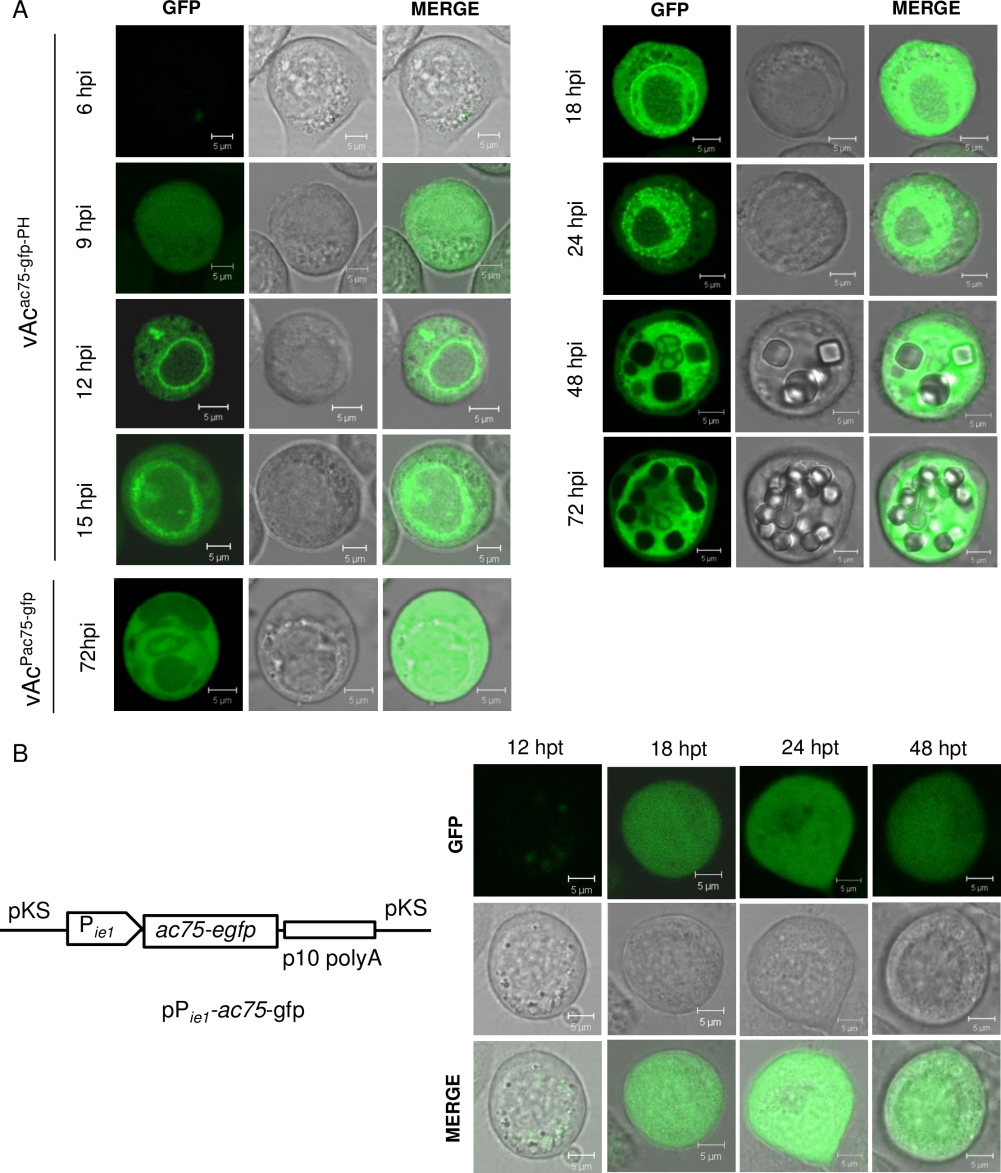
Subcellular localization of the AC75 in infected or transfected Sf9 cells. A. Sf9 cells infected with vAc^ac75-gfp-PH^ or vAc^Pac75-gfp^ were subjected to confocal microscopy at various time points as designated. B. Distribution of the AC75 in Sf9 cells transfected with pPie1-ac75-gfp, in which a copy of *ac75* coding sequence was fused with *egfp* at 3’-end and was placed under control of an *ie1* promoter. Transfected Sf9 cells were subjected to confocal microscopy at 12, 18, 24, and 48 hpt, respectively.

To monitor the subcellular localization of AC75, Sf9 cells infected with vAc^ac75-gfp-PH^, in which a copy of EGFP-coding sequence was fused with an *ac75* gene with its native promoter was inserted into the *polh* locus of the *ac75* knockout bacmid (Fig 2A), were examined by a confocal laser scanning microscope. For comparison, another AcMNPV recombinant vAc^Pac75-gfp^ that contains a copy of *egfp* under control of an *ac75* promoter was also used to infect Sf9 cells in same way. As shown in Fig 8A, light green fluorescence emitted by AC75-EGFP fusion protein was first observed in the cells as early as 6 hpi. At 9 hpi, fluorescence enhanced and spread almost evenly in the cytoplasm and the nucleus. At 12 hpi, strong fluorescence was distributed evenly in cytoplasm; fluorescence formed a bright ring along the nuclear membrane; the nucleus was filled with light blocky cloud-like fluorescent spots; a few bright fluorescent fragments and punctuate spots were seen in the cytoplasm. At 15 hpi and 18 hpi, condensed fluorescence formed a ring zone at the peripheral region of nuclei, although a large amount of AC75-EGFP still distributed throughout the cytoplasm. At 24 hpi, the fluorescent ring zone filled with condensed small fluorescent spots and became enlarged toward center of the nucleus. Only light fluorescence remained in the cytoplasm. A few small condensed fluorescent punctate spots were still observed in the cytoplasm. By 48 hpi, the condensed fluorescence ring zone disappeared, and the fluorescence spread almost evenly throughout the nucleus. A few OBs that did not emit fluorescence appeared in the nucleus that became enlarged at this time point. Some irregular fluorescent bodies aggregated together. These might be growing OBs. By 72 hpi, fluorescence was viewed evenly in the nucleus, and more OBs were present. In the cells infected by vAc^Pac75-gfp^, fluorescence emitted by EGFP spread evenly throughout the cells.

To determine if the transport of AC75 into the nucleus relies on other viral factors, a reporter plasmid containing the *ac75-egfp* chimera put under the control of an *ie1* promoter was transfected into Sf9 cells (Fig 8B). Under confocal laser scanning microscope, green fluorescence emitted by AC75-EGFP spread evenly throughout the cells transfected, at all time points (Fig 8B). This indicates that AC75 could spread into the nucleus freely. Its aggregation in the nucleus of the infected cells may be simply due to its continuous assembling into the virions or binding to other intranuclear components.

### AC75 co-immunoprecipitates with viral envelope protein E25

**Fig 9:**
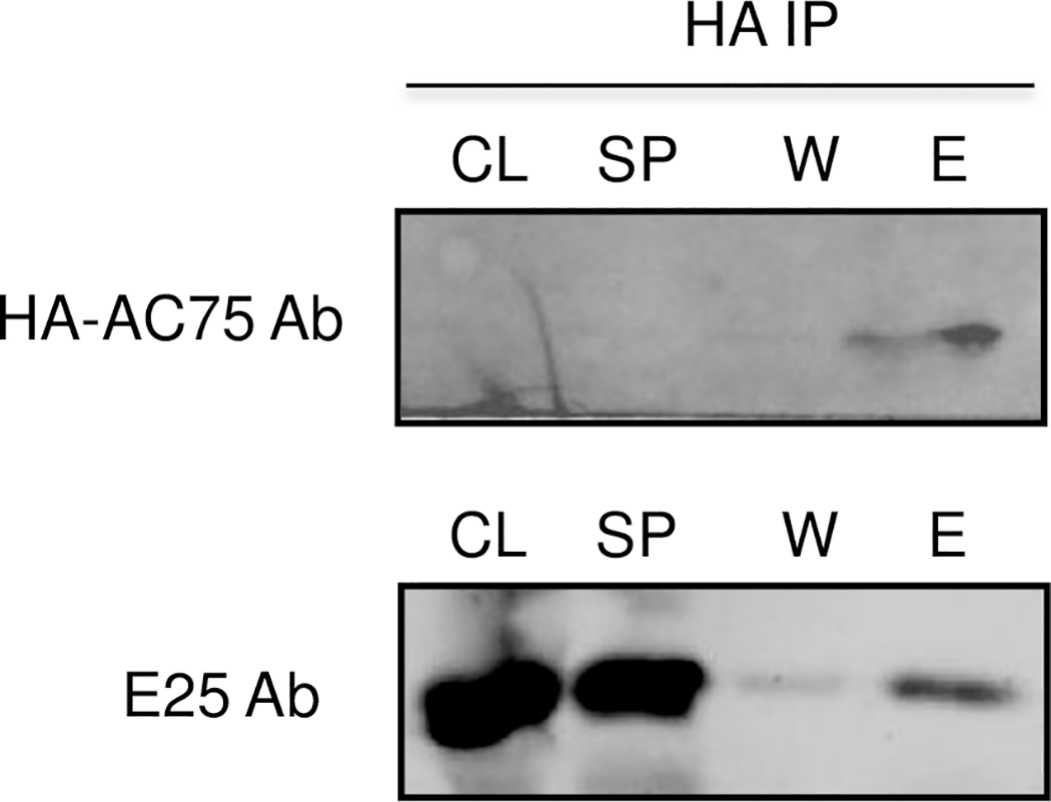
Co-immunoprecipitation of AC75 and ODV-E25. Extracts of Sf9 cells infected by vAc^ac75-HA^ were immunoprecipitated with anti-HA monoclonal antibody. The immunoprecipitates were analyzed by SDS-PAGE and western blot assays. HA-AC75 and E25 were detected with HA-specific monoclonal antibody and E25-specific polyclonal antibodies (Ab) respectively. CL, cytolysates; SP, supernatant; W, wash out; E, eluate.

To explore the mechanism that *ac75* used to influence egress and envelopment of nucleocapsids, immunoprecipitation assays were performed to detect interaction between AC75 and other viral proteins. The proteins selected for examination are BV and ODV envelope proteins E25 and E18 [3], and the tegument protein GP41. An AcMNPV recombinant expressing HA-tagged AC75 was constructed and used to infect Sf9 cells. The extracts of the infected cells were immunoprecipitated with HA-tag-specific monoclonal antibody. As shown in Fig 9, E25-specific antibodies detected a band of 25 kDa on western blots of the immunoprecipitates, while polyclonal antibodies specific to E18 or GP41 did not detect any bands of the corresponding proteins (data not shown).

## Discussion

In this study, AcMNPV AC75 was determined to be a structural protein associated with the nucleocapsid of BV and both of the nucleocapsid and envelope of ODV. This result is similar to the description for the homologs of AC75, HA69 in HearNPV, and BM61 in BmNPV, although BM61 was not determined to be envelope or nucleocapsid associated and HA69 was localized only to the nucleocapsid of ODV and between the envelope and nucleocapsid of BV [5, 7]. However, it was not been identified as a viral structural component in previous proteomics analyses of AcMNPV ODV and BV [3, 4].

In order to investigate the role *ac75* plays in the replication cycle of the virus, multiple AcMNPV mutants with *ac75* deleted or with *ac75*-knockout repaired were constructed and characterized for BV production, DNA replication, late gene expression and morphogenesis. Comparative analysis of the *ac75*-knockout and *ac75*-knockout repaired mutants and/or the wild types revealed that no BV was produced by the *ac75*-knockout mutants in transfected cells, whereas BV productivity of the *ac75*-knockout repaired mutant was similar to the wild type, indicating that *ac75* is essential for replication of the virus. Electron microscopy revealed the presence of VS, nucleocapsids, and OBs in the cells transfected by the *ac75* knockout bacmid vAc^ac75ko-PH^. However, the nucleocapsids were limited to nuclei and were unenveloped and the OBs did not contain any virions or nucleocapsids. Notably, the virus-induced intranuclear microvesicles that are precursor of the envelope of ODVs were absent in the transfected cells. This suggests that AC75 is required for formation of the *de novo* intranuclear microvesicles. The absence of the intranuclear microvesicles would subsequently block envelopment of nucleocapsids and production of ODV. Similar phenotypic changes were also observed in earlier studies on *ac76* and *ac93* [18–20]. These two genes were also shown to be required for formation of the *de novo* intranuclear membrane and ODV. Both AC76 and AC93 are associated with the envelope of BV and ODV. In addition, AcMNPV mutants lacking *ac66*, *ac78* or *ac103* were also shown to be defective in envelopment of ODV [21–23]. AC66 and AC78 are associated with both BV and ODV. AC66 is a component of the nucleocapsid while AC78 is an envelope protein.

It is known that BV and ODV acquire their envelopes by different mechanisms. The envelope of BV is from modified cytoplasm membrane while the envelope of ODV originates from the *de novo* intranuclear membrane. However, the knockout of *ac75* not only blocks envelopment of the nucleocapsids within the nucleus, but also eliminates production of BV. In the cells transfected with vAc^ac75ko-PH^, no nucleocapsids were found to be released from the nucleus or budding through the nuclear membrane (Fig 6). A similar phenomenon was also described for *ac76* and other genes mentioned above. In these cases, the absence of the *de novo* intranuclear membrane microvesicles resulting from deletion of the individual viral genes coincided with nucleocapsids that were not enveloped and were not released from nuclei [19–23]. In the cells infected/transfected by wt AcMNPV or specific gene-knockout repair mutants, nucleocapsids bud through the nuclear membrane and are enveloped [9, 14, 24]. The *de novo* intranuclear membrane microvesicles are considered to be derived from the inner nuclear membranes that have been modified with virally encoded ODV-specific envelope proteins [25]. The mechanism by which the nucleocapsids bud though the nucleus membrane is still unclear. We hypothesize that modification of the nuclear membranes is also essential for budding of the nucleocapsids through the nuclear membrane in addition to formation of intranuclear membrane microvesicles. AC75, AC76 and AC93 may be involved in modification of inner nuclear membranes. Some proteins on the surface of nucleocapsids may be responsible for recognition of and interaction with the modified inner nuclear membranes. Missing of a critical protein involved in either modification of the inner nuclear membrane or recognition of the modified inner nuclear membrane would block formation of the *de novo* intranuclear membrane microvesicles and/or egress of the nucleocapsids from the nucleus.

Confocal microscopy revealed that AC75-EGFP expressed by the *ac75* reporter virus vAc^ac75-gfp-PH^ formed a ring zone in the periphery of the nucleus in infected cells, from 15 to 24 hpi (Fig 8A). The region is the site of intranuclear envelopment of nucleocapsids to produce ODVs [26]. The nucleocapsids are assembled in the VS of the nucleus and then transported to the periphery of the nucleus to acquire an envelope or to bud through the nuclear membrane. Previous reports showed that AcMNPV E25, E26, AC76, AC92, AC109, and AC132 also localized to the ring zone in the periphery of the nuclei in infected cells [9, 10, 19, 27–29]. E25, E26, AC76 and additional viral envelope proteins E18, ODV-E56, and ODV-E66 were found to be associated with the intranuclear microvesicles [30–33]. AC132 was shown to interact with E18 that is associated with the envelopes of both BV and ODV and it was suggested to be involved in envelopment of ODV [9]. In this study, AC75 was found to interact, either directly or indirectly, with another envelope protein E25 common to BV and ODV. This suggests that AC75 may be directly involved in the envelopment of nucleocapsids, in addition to being associated with formation of the intranuclear microvesicles. In addition, AC75-EGFP was also found in the center of the nucleus with blocky cloud-like distribution (Fig 8A). As a structural protein, AC75 is likely assembled into nucleocapsids in this region. The fluorescent ring along the nuclear membrane observed at 12 hpi might comprise the nucleocapsids labeled by AC75-EGFP budding through the nuclear membrane since AC75 is associated the nucleocapsids. The bright fluorescent fragments and punctate spots present in the cytoplasm might be the progeny nucleocapsids released from nuclei, some of which are contained in large vesicles [9, 14, 24].

The data from this study suggests that AC75 is an important structural protein that is required for formation of *de novo* intranuclear membrane microvesicles, envelopment of ODV and egress of nucleocapsids from the nucleus. It may be involved in nucleocapsid envelopment by interacting with envelope protein E25. Based on the data from this study and the similar phenotypes observed from the AcMNPV mutants lacking *ac76*, *ac93*, *ac66*, *ac78* or *ac103*, we hypothesize that the envelope of the nucleocapsids released from nuclei and the *de novo* intranuclear membrane microvesicles required for envelopment of ODV originated from the same modified inner nuclear membranes. The same set of viral proteins may affect both egress of nucleocapsids from the nucleus and formation of the intranuclear microvesicles by involving modification of nuclear membrane in infected cells.
